# Metatranscriptomics and Pyrosequencing Facilitate Discovery of Potential Viral Natural Enemies of the Invasive Caribbean Crazy Ant, *Nylanderia pubens*

**DOI:** 10.1371/journal.pone.0031828

**Published:** 2012-02-27

**Authors:** Steven M. Valles, David H. Oi, Fahong Yu, Xin-Xing Tan, Eileen A. Buss

**Affiliations:** 1 Center for Medical, Agricultural and Veterinary Entomology, Agriculture Research Service, United States Department of Agriculture (USDA-ARS), Gainesville, Florida, United States of America; 2 Interdisciplinary Center for Biotechnology Research, University of Florida, Gainesville, Florida, United States of America; 3 SeqWright, Houston, Texas, United States of America; 4 Department of Entomology and Nematology, University of Florida, Gainesville, Florida, United States of America; INRA, France

## Abstract

**Background:**

*Nylanderia pubens* (Forel) is an invasive ant species that in recent years has developed into a serious nuisance problem in the Caribbean and United States. A rapidly expanding range, explosive localized population growth, and control difficulties have elevated this ant to pest status. Professional entomologists and the pest control industry in the United States are urgently trying to understand its biology and develop effective control methods. Currently, no known biological-based control agents are available for use in controlling *N. pubens*.

**Methodology and Principal Findings:**

Metagenomics and pyrosequencing techniques were employed to examine the transcriptome of field-collected *N. pubens* colonies in an effort to identify virus infections with potential to serve as control agents against this pest ant. Pyrosequencing (454-platform) of a non-normalized *N. pubens* expression library generated 1,306,177 raw sequence reads comprising 450 Mbp. Assembly resulted in generation of 59,017 non-redundant sequences, including 27,348 contigs and 31,669 singlets. BLAST analysis of these non-redundant sequences identified 51 of potential viral origin. Additional analyses winnowed this list of potential viruses to three that appear to replicate in *N. pubens*.

**Conclusions:**

Pyrosequencing the transcriptome of field-collected samples of *N. pubens* has identified at least three sequences that are likely of viral origin and, in which, *N. pubens* serves as host. In addition, the *N. pubens* transcriptome provides a genetic resource for the scientific community which is especially important at this early stage of developing a knowledgebase for this new pest.

## Introduction

Metagenomic analysis [1] coupled with new generation sequencing technologies have revolutionized the way in which entomologists can search for potential pathogens for use as insect biological control agents. Historically, discovery of pathogens of insects relied upon arduous explorations for unhealthy or dying insects followed by the identification and isolation of the microbe(s) responsible; a process that often took years to complete [2], [3]. Metagenomic analysis not only reduces discovery time to days, but it is also capable of identifying numerous pathogenic organisms simultaneously. Indeed, metagenomic analysis may even be utilized to examine insects retrospectively from archived specimens. A number of sequencing projects of environmental samples of insects have demonstrated successful discovery of viruses that show promise as insect control agents [4], [5], [6], [7], [8].

*Nylanderia pubens* (Forel), previously *Paratrechina pubens* [9], is an invasive ant species that in recent years has developed into a serious pest problem in the Caribbean and United States [10], [11]. A rapidly expanding range, explosive localized population growth, and control difficulties have elevated this ant to pest status. Professional entomologists and the pest control industry in the United States are urgently trying to understand its biology and develop effective control methods [12], [13], [14]. Efforts have primarily focused on pursuing development of insecticide-based control strategies [15], as well as the effort presented here to identify self-sustaining, biological control agents specific to *N. pubens*. While viruses can be important biological control agents against pest insect populations [16], none are known to infect *N. pubens*. Therefore, the objective of this research was to employ a metagenomics approach coupled with 454-based sequencing technology to examine the *N. pubens* transcriptome for viral infections based on sequence homology/identity with known viral sequences. The ultimate goal is the exploitation of viral discoveries as biologically-based agents for controlling *N. pubens*. As an added benefit, transcriptome sequencing provides a genetic resource for the scientific community which is especially important at this early stage of developing a knowledgebase for *N. pubens*.

## Methods

### Ants

*N. pubens* colonies were obtained from field sites located in Desoto (April, 2011), Hillsborough (April, 2011), Alachua (March through May, 2011), and Duval (March, 2011) counties in Florida and subsequently maintained in the laboratory. No specific permits were required to collect these field specimens because they did not occur in locations protected in any way. The field collections did not involve endangered or protected species. Colonies were reared separately in nesting tubes described by Oi and Williams [17] and fed frozen crickets, live housefly larvae, 10% sucrose solution, and water. Identifications of ants were made based on characters listed in Trager [18] and LaPolla et al. [9]. However, there is uncertainty regarding species assignment of these ants currently being reported from Florida, Texas, Louisiana and Mississippi based on morphometric and DNA sequence data. Nonetheless, the current consensus regards these invasive ants as the same species (D. Gotzek, personal communication). Representative voucher specimens were collected and retained in 95% ethanol at the USDA-ARS, Center for Medical, Agricultural and Veterinary Entomology, Gainesville, Florida.

### mRNA extraction, purification, library construction, and sequencing

Total RNA was extracted from samples of colonies of *N. pubens* by the Trizol (Invitrogen, Carlsbad, CA) method according to the manufacturer's instructions. Samples were taken from nine colonies. A total of 609 ants of different life stages (workers, alates, queens, larvae, pupae, and eggs) were used to prepare the total RNA. RNA quality of each preparation was assessed by microfluidic analysis on an Agilent 2100 Bioanalyzer (Agilent, Cary, NC) using the RNA 6000 Nano kit according to the manufacturer's directions. Microfluidic assays were completed immediately after RNA extraction using a 1 µl volume of purified sample. RNA samples of acceptable quality were pooled and used as source material for mRNA purification. mRNA was isolated from the total RNA sample using the Oligotex mRNA Mini Kit (Qiagen, Valencia, CA) following the manufacturer's instructions. The isolated mRNA was then utilized to prepare a non-normalized fragment library suitable for 454 platform sequencing using the NEBNext mRNA Sample Pre Reagent Set 2 (New England BioLabs, Ipswich, MA) following the manufacturer's protocol. The library was used as template for emulsion PCR using the GS Titanium LV emulsion PCR Kit (Lib-L; Roche, Manheim, Germany) following the manufacturer's instructions. DNA beads generated from the emulsion PCR reactions were used for Titanium plate 454 sequencing, using the GS Titanium Sequencing Kit XLR70 (Roche). *De novo* assembly was performed for the generated sequencing data using the Newbler software (Roche).

### Bioinformatic analysis

An initial assembly of the sequences was performed with Newbler Assembler Version 2.3 (454 Life Science, Branford, CT), employing masking and trimming sequencing repeats, primers and/or adaptors used in cDNA library preparation. These hybridized sequences (contigs and leftover singletons) were further assembled with Paracel Transcript Assembler version 3.0.0 (PTA; Paracel Inc., Pasadena, CA).

In PTA, all sequences were masked for universal and species-specific vector sequences, adaptors, and PCR primers used in cDNA library creation. *Escherichia coli* contamination and mitochondrial and ribosomal RNA genes were identified and removed from input sequences using default settings to ascertain the novelty of the sequences. The poly (A/T) tails and intrinsic repeats, such as simple sequence repeats and short interspersed elements (SINE), were annotated prior to clustering and assembly. Low base-call quality data were trimmed from the ends of individual sequences and sequences <75 bp were excluded from consideration during initial pair-wise comparisons. After cleanup, sequences were passed to the PTA clustering module for pair-wise comparison and then to the CAP3-based PTA assembly module for assembly. The PTA assembly was performed based on the sequences of the contigs and the leftover singletons generated from the Newbler assembly.

Large-scale homology database searches of the PTA sequence data set were conducted against the National Center for Biotechnology Information (NCBI) NR and NT databases using BLAST (blastx and blastn) [19] with an in-house computational pipeline. To obtain a more accurate and complete description of potential gene function for each queried sequence, the top 100 BLAST hits were retrieved. Sequences with the best scoring BLAST hit (≤1e^−5^) and the corresponding gene ontology (GO) classification were annotated to the queried sequence [20]. GO term assignments were binned according to the categories, biological processes, cellular components, and molecular functions. BLAST results and GO term assignments were completed in BlastQuest, an SQL database developed by the Interdisciplinary Center for Biotechnology Research, University of Florida, that facilitates similarity-based sequence annotation with gene ontology information [21]. In addition, the sequences were characterized with respect to functionally annotated genes by BLAST searching against NCBI specific reference sequences (RefSeq) for *Homo sapiens* (38,556 sequences), *Drosophila* (21,099 sequences) and Formicidae (74,540 sequences). Queries were considered to have a clear homolog of the searched organism when e-values were ≤1e-4, the length of the aligned segment was ≥50 bp, and identity >85%, which essentially eliminated spurious hits while preventing elimination of medium-sized proteins.

### Data Availability

Raw 454 reads and assembled contigs were deposited in the NCBI database. The *N. pubens* sequence data are publicly available and accessible through the NCBI website accession numbers Ant_454Assem_NCBI.sqn: JP773711 - JP820231.

### Characterization of ESTs with viral identity

**Figure 1:**
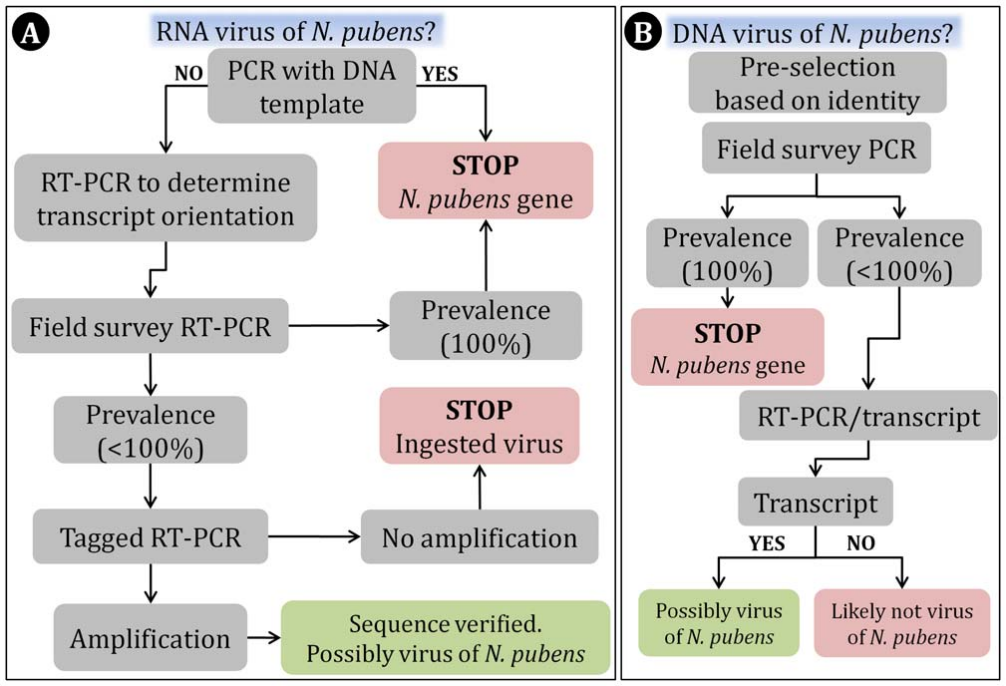
Stepwise decision tree employed as a guide to assess the likelihood that a given sequence was of RNA (A) or DNA (B) viral origin.

Sequences identified as exhibiting significant viral homology/identity were selected from the *N. pubens* annotated list and further evaluated in an attempt to establish their origin—viral, host, or otherwise. Evaluations were also conducted to ascertain whether identified viral sequences were simply being ingested by the ants or were replicating (i.e., *N. pubens* was serving as host). Figure 1 summarizes the step-by-step decision tree employed to determine the likelihood that a given EST was of viral origin. Note that the process illustrated in figure 1 served only as a general guide to identify sequences of non-viral origin and non-replicating (e.g., ingested) viral sequences. Based on previous studies [5], [7], this winnowing method significantly improves the virus discovery process.

### RNA virus confirmation

Oligonucleotide primers were designed to each EST with significant (e-score ≤1e-4) identity to the RNA viral sequences (Table 1). PCR was conducted with RNase-treated DNA extracted from the same *N. pubens* colonies used in expression library creation. If PCR yielded an amplicon, it was concluded that the sequence was likely an *N. pubens* gene and experiments concerned with this sequence were terminated because all of the RNA virus sequences exhibited identity with single-stranded RNA viruses that do not integrate in their host genome (no DNA stage generated). Sequences not generating an amplicon by PCR were next evaluated by RT-PCR. Ants from field-collected colonies were evaluated by RT-PCR for the presence of sequence template. If amplification was observed in 100% of the samples, it was assumed that the sequence was of host (or other) origin and further experiments with the sequence were terminated. Viral infections rarely exhibit an incidence of 100% among field-collected arthropods [22]. If amplification was observed in less than 100% of the samples, tagged-RT-PCR was conducted with the appropriate oligonucleotide primers to detect the replicating genomic strand [23]. This method permits discrimination of each genome strand without carryover effects causing false positive detection of either strand [23]. Tagged-RT-PCR employed the use of the appropriate oligonucleotide primer (Table 1) appended at the 5′ end with a TAG sequence (5′GGCCGTCATGGTGGCGAATAA) that was used in a cDNA-synthesis reaction (forward primer for positive strand viruses and reverse primer for negative strand viruses). Two-step RT-PCR was employed to amplify a portion of the genome strand. First, 1 µl (50 ng) of total RNA was mixed with 10 mM dNTPs, 1 µM of the appropriate tagged oligonucleotide primer, heated to 65°C for 5 minutes, and then placed on ice for at least 1 minute. First strand buffer and Superscript reverse transcriptase (RT, Invitrogen) were then added and the reaction mixture was incubated at 55°C for 1 hour before inactivating the RT at 70°C for 15 minutes. Unincorporated oligonucleotides were digested with 10 units of Exonuclease I (New England Biolabs, Ipswich, MA) at 37°C for 1 hour. The reaction was terminated by heating to 80°C for 20 minutes.

**Table 1:** Oligonucleotide primers designed to each corresponding *N. pubens* sequence with viral identity.

Designation	Forward oligonucleotide primer (5′→3′)	Reverse oligonucleotide primer (5′→3′)
Assem.6302.C1	***p1179*** TCGTCTTCCAGCGTCATCTGATGATCTA	***p1180*** AGGATATAAATCAGGGAAACACATGACATCCA
G49287O02I5H2T	***p1255*** CCAAGCAAGAATCTGAACTTCAGCATCTTG	***p1256*** GTGGGCTAACCTGCAACGCGG
G49287O02FSJQT	***p1253*** CCAAGCAAGAATCTGAACTTCAGCATCTTG	***p1254*** ACCTGCAACGCGGCATGGATC
49287O01EPUMA	***p1251*** GTTTGTTCGAAACATTGCCCATCAATT	***p1252*** TATGCTTGAGAAGAGACGTTTCTAAGCTCGAGA
G49287O01A0XGN	***p1249*** ACACCTGAACCGCCGTACTTTGCAC	***p1250*** TCGGTTCAGGTTCGCTACACGCGA
G49287O02GNUGU	***p1247*** ACCACGACGAAGCACAAATTGAAGTT	***p1248*** ATTAGTGACAGTTTGCCCAACTTGTTCCA
G49287O01BKDVJ	***p1245*** ATGATAGACAGTTTCCATGCGCTACTCGA	***p1246*** TTTGTATATTTGCATTACGACTACCTTCATGAGA
Assem.15438.C1	***p1243*** TCAGTATGTTCCAGTGGGCCGGACA	***p1244*** GGTCGTGGCAGTCAAAGCCGAGGA
Assem.10577.C1	***p1239*** TACGATTGAAGTAATAGATAAAAGCATAGCGAA	***p1240*** AACTCTCACTATTCTTTGGTGCATCATCTT
Assem.3776.C1	***p1167*** CCCTACTGACTGACGAACAGATTGCTTC	***p1168*** TGTTGTTGAGCGTAATGAGTCCGTCCT
Assem.2829.C1	***p1181*** TCCAGTGAGAATATGCATAGCCTAAGACTCCA	***p1182*** CAGCCTCACAAAATCTAACAGAATCGGA
Assem.13129.C1	***p1241*** ACTGACTAGCTTCCCTAGGAGTAGGTTGAGCTTA	***p1242*** TCTGATCCCAAGGTCCTCTCCAATCTT
Assem.13541.C1	***p1257*** CTCCTGAACTTATATCCTCCGTATTAAGTGATCA	***p1258*** TGTCCAAAGATAATTCGTCATCAATCATAGTAA
Assem.13287.C1	***p1169*** ACTTCACTTGTATATGGAGATCCCTCCATACAA	***p1170*** TTGCTTCGTGATATGTCATTCCTGGATACAAT
Assem.8702.C1	***p1172*** TGGTACTGGTATGTCGGATGTGATGAGCT	***p1171*** TGAGGTCTTGACACTGGTAGTGTTGAAATGA
G49287O01APTQA	***p1259*** ATCAAGGAATCTTGCAGAAGCTCTGGACTAT	***p1260*** AGCACGTGCTGATATTTTGATTCCACCT
Assem.4695.C1	***p1197*** AGATGATGAAGCACTGCACCAGTTTTC	***p1198*** GCTAATAGATAATTCTGATATTGGGTTAGAATCAGT
Assem.19410.C1	***p1199*** AGATAGGAGAGTGAGTCTTAATTGAAAAGATAAGA	***p1200*** GGGTTTCACTTTGGCAAATTTGATGT
Assem.16207.C1	***p1191*** AGAGGAATGAATTAATCAATGGTGACATGAGA	***p1192*** ACAGTCTTTAATTTCACCCTTTAGCCATGGTA
Assem.13720.C1	***p1193*** GTGGATAGGTCTGATATTGCTGGAGTGAG	***p1194*** ATCTCGTCCATATCAGTTAAGGAAACGAAC

PCR was subsequently conducted in a 25 µl volume containing 2 mM MgCl_2_, 200 µM dNTP mix, 0.5 units of Platinum *Taq* DNA polymerase (Invitrogen), 0.2 µM of each oligonucleotide primer, and 5 µl of the cDNA preparation. PCR products were separated on a 1% agarose gel and visualized by SYBR-safe (Invitrogen) staining. If the replicating strand of the virus was not detected, it was assumed the ant was not serving as host to the putative virus (e.g., may have simply been ingested) and further experiments with the sequence were terminated. If the replicating strand was detected by tagged-PCR, the amplicon was cloned and its sequence verified by Sanger sequencing. In these instances, sequences were considered to be likely from a virus infecting *N. pubens* and for the purposes of this study further experiments were terminated.

### DNA virus confirmation

Field-collected *N. pubens* colonies (Alachua and Hillsborough counties, Florida) were evaluated by PCR for the presence of sequence template. If amplification was observed in 100% of the samples, it was assumed that the sequence was of host origin and further experiments with the sequence were terminated. If amplification was observed in less than 100% of the samples, attempts were made to detect the presence of a corresponding transcript by RT-PCR. If a transcript was detected the sequence was verified by Sanger sequencing and considered a likely gene from a virus infecting *N. pubens*.

## Results and Discussion

### 454 sequencing data assembly and annotation

**Figure 2:**
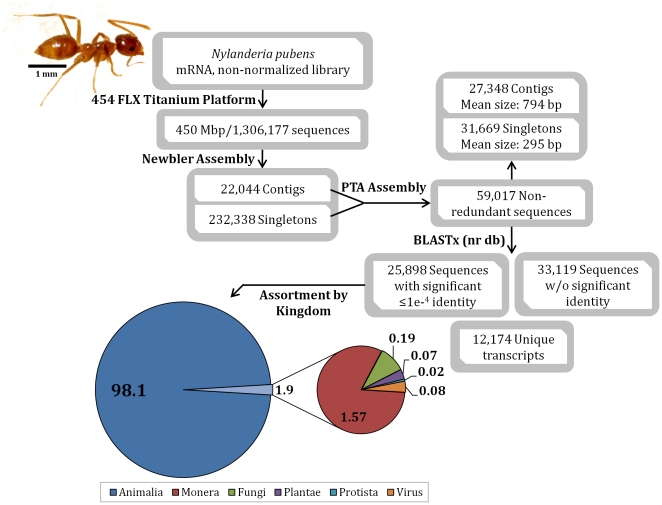
Diagram summarizing the work flow and results for expression library creation, sequencing, assembly and kingdom assignments for the Caribbean crazy ant, *Nylanderia pubens* (top left).

The single production GS-FLX Titanium 454 platform sequencing run (two half plates) of the non-normalized *N. pubens* expression library generated 1,306,177 raw sequence reads comprising 450 Mbp (Fig. 2). *De novo* assembly of the raw data with Newbler yielded 22,044 contigs and 232,338 singletons. Subsequent assembly by PTA resulted in generation of 59,017 non-redundant sequences, including 27,348 contigs (average size 794 bp) and 31,669 singlets (average size 295 bp). Among these sequences, 27.9% (16,458) were greater than 500 bp and 72.1% (42,533) were greater than 300 bp. BLASTX analysis of these non-redundant nucleotide sequences identified 25,898 (43.9%) with significant (e-value≤1e^−4^) similarity and 33,119 (56.1%) returned no significant similarity. A significant percentage (47%) of the gene sequences (12,174) identified were found to be unique to *N. pubens*.

**Figure 3:**
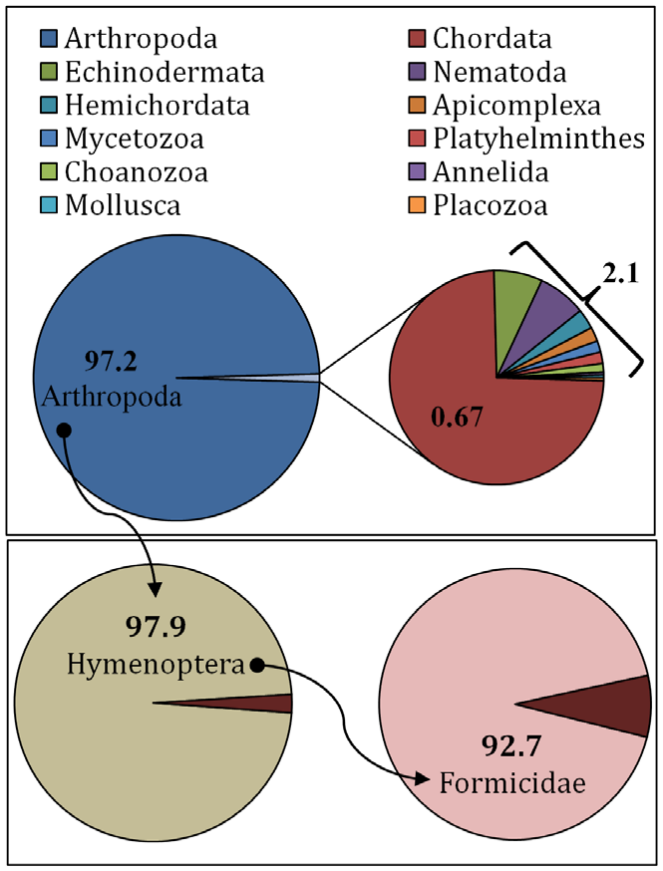
Distribution by phyla of *N. pubens* sequences with significant (expectation score ≤1e^−4^) BLAST identity (upper panel). Sequences with arthropod identity were further categorized by order (left, lower panel). Lastly, sequences with hymenoptera identity were further categorized by family (right, lower panel). All values are shown as percentages.

When categorized taxonomically based on the significant (expectation score ≤1e^−4^) BLAST results, 98.1% (25,405) of the *N. pubens* annotated sequences exhibited identity with genes from Animalia (Fig. 2). The remaining 1.9% of the sequences exhibited identity with known genes from Monera (1.6%), Fungi (0.19%), Plantae (0.07%), Protista (0.02%), and Viruses (0.08%). Further analysis showed that 97.2% of the annotated sequences exhibited identity with genes from organisms within the phylum Arthropoda. Subsequent categorization (Fig. 3) of these sequences showed that 97.9% of the Arthropoda genes exhibited identity to Hymenoptera (bees, wasps, and ants) and among these, 92.7% to Formicidae (ants). Among the sequences that showed identity to ant genes, the majority (70.5%) exhibited identity with ants within the subfamily, Formicinae (specifically, *Camponotus floridanus*). The remaining sequences with identity to ants included the subfamilies Ponerinae (12.8%) and Myrmicinae (16.7%). Thus, the majority of the annotated *N. pubens* sequences yielded identity with its taxonomically nearest neighbor represented in the Genbank database, specifically, *Camponotus floridanus* [24]. *N. pubens* and *C. floridanus* are both members of the Formicinae.

**Figure 4:**
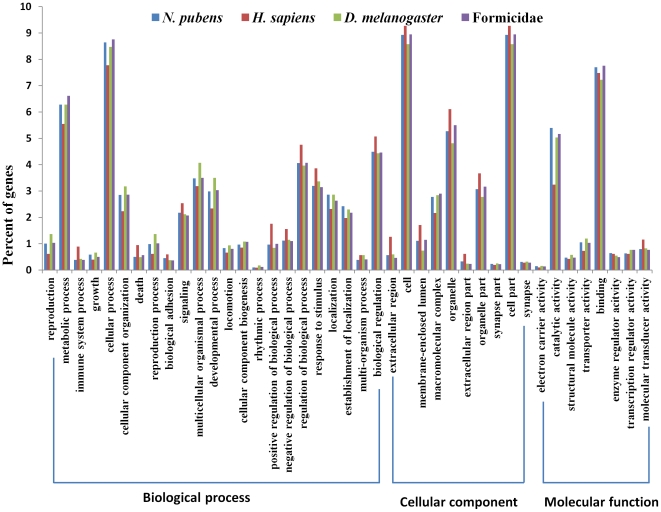
*N. pubens* sequences annotated with a gene ontology term (GO) and compared with reference sequences of *Homo sapiens*, *Drosophila melanogaster*, and Formicidae.

Categorization of each *N. pubens* annotated sequence (*n* = 25,898) returning a known function was summarized by assigning a gene ontology (GO) term when available (biological processes, cellular components, or molecular functions). GO analysis was also completed for reference sequences of *Homo sapiens*, *Drosophila melanogaster*, and Formicidae for comparison. The distribution of *N. pubens* genes among GO categories was essentially similar regardless of taxon (Fig. 4). In each of the three main GO categories, metabolic process, cellular process, cell, cell part, and binding terms were dominant (each category represented >5% of the annotated gene functions). Also well represented were proteins involved in regulation of biological process, biological regulation, catalytic activity, and organelle (each representing 3.5 to 5% of the annotated gene functions). As expected, the most significant departures were observed between *Homo sapiens* and *N. pubens* GO categories. Conversely, the gene representation was more consistent among *Drosophila melanogaster*, Formicidae, and *N. pubens*.

### Viral discovery

BLAST analysis of the 59,017 non-redundant sequences yielded from the *N. pubens* library resulted in the identification of 51 sequences of putative viral origin (Table S1 and Table 2). Among them, 31 sequences did not meet the threshold for significance (an expectation score >1e^−4^) and were not examined further to establish their source, viral, host, or otherwise (Table S1). However, despite expectation scores greater than 1e^−4^, some of these sequences could represent viruses that infect *N. pubens*. Indeed, in a similar study, putative viral sequences from an expression library from the red imported fire ant, *Solenopsis invicta*, which were not considered significant by BLAST analysis, were ultimately found to be of viral origin [5], [7]. Database underrepresentation or distant relationships may contribute to a non-significant BLAST expectation score. Among the *N. pubens* sequences with BLAST expectation scores greater than 1e^−4^ (Table S1), 24 indicated identity with DNA viruses and 7 to RNA viruses. Many of the putative DNA (41.9%) and RNA (71.4%) viral genes were from virus families with a host range that does not normally include arthropods.

**Table 2:** *N. pubens* transcriptome sequences yielding a significant (≤1e^−4^) expectation score from BLAST analysis and exhibiting viral identity.

Designation	Size	E-value	Gene	Virus	Genome	Family	Host range
Assem.6302.C1	954	6.05e^−66^	Hypothetical protein 4	Hyposoter didymator virus	dsDNA	Polydnaviridae	Invertebrates
49287O01EPUMA	335	1.40e^−20^	Hypothetical protein 3	Hyposoter didymator virus	dsDNA	Polydnaviridae	Invertebrates
G49287O01BKDVJ	483	1.14e^−14^	Hypothetical protein 5	Hyposoter didymator virus	dsDNA	Polydnaviridae	Invertebrates
G49287O02I5H2T	329	1.04e^−31^	Virion structural protein	Myxococcus phage Mx8	dsDNA	Podoviridae	Bacteria
G49287O02FSJQT	329	1.04e^−31^	Virion structural protein	Myxococcus phage Mx8	dsDNA	Podoviridae	Bacteria
G49287O01A0XGN	520	2.66e^−20^	Hypothetical protein	Listonella phage phiHSIC	dsDNA	Siphoviridae	Bacteria
G49287O02GNUGU	335	1.23e^−16^	DNA polymerase	Pseudomonas phage	dsDNA	Myoviridae	Bacteria
Assem.15438.C1	503	1.35e^−07^	Hypothetical protein	Vibrio phage	dsDNA	Myoviridae	Bacteria
Assem.10577.C1	392	2.03e^−07^	Hypothetical protein	Aeromonas phage	dsDNA	Myoviridae	Bacteria
Assem.2829.C1	1628	1.25e^−37^	RNA polymerase	Uukuniemi virus	ss(−)RNA	Bunyaviridae	Vertebrates, plants
Assem.13129.C1	490	3.99e^−15^	RNA polymerase	Uukuniemi virus	ss(−)RNA	Bunyaviridae	Vertebrates, plants
Assem.16207.C1	711	1.32e^−06^	RNA polymerase	Gouleako virus	ss(−)RNA	Bunyaviridae	Vertebrates, plants
Assem.13541.C1	395	1.17e^−14^	L protein	Strawberry crinkle virus	ss(−)RNA	Rhabdoviridae	Vertebrates, plants
G49287O01APTQA	427	6.82e^−14^	Polymerase	Iranian maize mosaic nucleorhabdovirus	ss(−)RNA	Rhabdoviridae	Vertebrates, plants
Assem.19410.C1	413	8.79e^−07^	Polymerase	West Caucasian bat virus	ss(−)RNA	Rhabdoviridae	Vertebrates, plants
Assem.3776.C1	1328	2.62e^−75^	Nonstructural polyprotein	Solenopsis invicta virus 3	ss(+)RNA	Unclassified	Invertebrates
Assem.13287.C1	570	2.18e^−14^	Polyprotein	Kelp fly virus	ss(+)RNA	Unclassified	Invertebrates
Assem.8702.C1	1282	3.48e^−14^	Nonstructural polyprotein	Solenopsis invicta virus 3	ss(+)RNA	Unclassified	Invertebrates
Assem.4695.C1	953	4.02e^−11^	RNA polymerase	Rice grassy stunt virus	ss(+)RNA	Unclassified	Plants
Assem.13720.C1	402	3.40e^−06^	Capsid protein	Nilaparvata lugens commensal X virus	ss(+)RNA	Unclassified	Invertebrates

Each sequence designation, its corresponding size, BLAST results (expectation value, gene and virus relatedness), and characteristics (nucleic acid composition, family and host range) are provided. DNA viruses are consolidated in the upper panel and RNA viruses in the lower panel.

Twenty sequences from the *N. pubens* expression library yielded significant BLAST expectation scores of putative viral origin (Table 2); nine sequences were similar to DNA and eleven to RNA virus sequences. Among the potential DNA virus sequences, six exhibited identity with bacterial-infecting phage in the *Podoviridae*, *Siphoviridae*, and *Myoviridae* and were excluded from further experimentation (Table 2). Three sequences exhibited strong similarity with protein genes 3, 4, and 5 of the polydnavirus, Hyposoter didymator virus. This finding could potentially represent the discovery of an ancestral polydnavirus. However, PCR conducted with oligonucleotide primers (Table 1) designed toward each of these sequences (Assem.6302.C1, 49287O01EPUMA, and G49287O01BKDVJ) resulted in amplification (of anticipated size) from all *N. pubens* field colonies examined (*n* = 8). Polydnaviruses are known to infect insect parasitoids in a limited number of subfamilies of the Braconidae and Ichneumonidae [25]. As these viruses integrate into the host genome, portions of the wasp's genome are thought to incorporate into the virus genome—most likely because they proved useful for successful parasitism [25], [26], [27]. Thus, although sequence identity suggests polydnaviral origin, it is more likely that these sequences represent related insect (specifically Hymenopteran) genes.

The remaining eleven sequences were related to genes of RNA viruses (Table 2); six negative and five positive sense, single-stranded RNA virus genes were identified. The negative-sense RNA viruses included six viruses from two families (*Bunyaviridae* and *Rhabdoviridae*) and the positive-sense virus genes were all related to unclassified viruses, four of which infect arthropods.

Sequences Assem.2829.C1, G49287O01APTQA, Assem.16207.C1, and Assem.13720.C1 were considered to be of non-viral origin because 100% of the *N. pubens* colonies examined from the field (*n* = 6 to 8) produced amplicons of anticipated size from a DNA template with oligonucleotide primers designed to each respective sequence, suggesting that they were likely to be part of the ant genome (Fig. 1). Conversely, oligonucleotide primers designed to sequences Assem.13541.C1, Assem.13129.C1, Assem.4695.C1, Assem.19410.C1, Assem.13287.C1, Assem.8702.C1, and Assem.3776.C1 failed to yield an amplicon from *N. pubens* DNA template.

*N. pubens* field colonies were examined for the presence of sequences Assem.13541.C1, Assem.13129.C1, Assem.4695.C1, and Assem.19410.C1 by RT-PCR. Oligonucleotide primer p1258 (but not p1257) designed to Assem.13541.C1 successfully produced cDNA and subsequently an amplicon of anticipated size by PCR in 100% of the *N. pubens* field colonies evaluated suggesting that this gene sequence was likely of ant origin (Table 3). Similar results were also observed for sequences Assem.13129.C1, Assem.4695.C1 and Assem.19410.C1. However, amplicons were generated from all field samples evaluated regardless of which oligonucleotide primer (p1241/p1242, p1197/p1198, p1199/p1200, respectively) was used for cDNA synthesis. These results suggest the presence of ambisense RNA template possibly of viral origin. Assem.19410.C1 exhibited identity with a bunyavirus (Table 3) that are known to contain ambisense RNA which could explain cDNA synthesis by both primers. However, viral prevalence of 100% would not be expected among field-collected samples of ants [22]. Furthermore, the *Bunyaviridae* are typically arthropod-borne and vectored to mammals by mosquitoes, ticks and sandflies [28]. Because *N. pubens* is an omnivorous ant species it would be expected to consume other arthropods and plants—a possible source of bunyaviruses. However, the diet of these ants has not been established.

**Table 3:** Proportion of *N. pubens* field-collected colony samples producing an amplicon with oligonucleotide primers (see Table 1) designed to each sequence indicated.

Sequence	PCR (DNA template) [*n*]	RT-PCR (RNA template) [*n*]	Tagged RT-PCR^a^ (RNA template) [*n*]
Assem.2829.C1	100 [8]	-	-
G49287O01APTQA	100 [8]	-	-
Assem.16207.C1	100 [6]	-	-
Assem.13720.C1	100 [6]	-	-
Assem.13541.C1	0 [8]	100 [6]	-
Assem.13129.C1	0 [11]	100 [8]^b^	-
Assem.4695.C1	0 [13]	100 [16]^b^	-
Assem.19410.C1	0 [13]	100 [16]^b^	-
Assem.13287.C1	0 [6]	66 [6]	7.7 [26]
Assem.8702.C1	0 [6]	66 [6]	2 [50]
Assem.3776.C1	0 [6]	50 [6]	7.7 [26]

In each column, the percentage of samples and the total number evaluated [*n*] yielding an amplicon are provided.

a For positive strand viruses, the forward tagged primer was used to detect the negative (or replicative) viral genome strand. Similarly, for negative strand viruses the reverse tagged primer was used to detect the positive (or replicative) viral genome strand.

b All (100%) of these field samples yielded an amplicon when either the forward or reverse oligonucleotide primer was used in cDNA synthesis suggesting the presence of ambisense RNA.

Examination of field colonies of *N. pubens* for sequence Assem.13129.C1 by RT-PCR also yielded an amplicon of anticipated size among 100% of the colonies examined (Table 3). Assem.13129.C1 exhibited identity with a rhabdovirus (Table 2). The genomes of viruses in this family contain inverted complementary sequence ends and up to 5% of the viral RNA population may be comprised of full-length positive strands [28]. These characteristics may explain why cDNA was able to be synthesized with both oligonucleotide primers. However, as with sequence Assem.19410.C1, an incidence of 100% would not be expected. Interestingly, Fort et al. [29] have recently shown that rhabdovirus genome fragments are widely integrated into their arthropod hosts. Although the origin of sequences Assem.19410.C1 and Assem.13129.C1 is not clear, based on the decision parameters established *a priori* (Fig. 1), the conclusion is that they are not likely from a virus replicating in *N. pubens*.

Assem.4695.C1 displayed identity with an unclassified positive-strand RNA virus; these viruses produce both genome strands (plus and minus) during their life cycles which could explain these results. However, copies of the minus strand are typically several hundred-fold less than the plus strand and are only present during periods of active replication [30], [31]. Therefore, the presence of both genome strands would not be anticipated among 100% of field-collected colonies.

The most promising virus leads appear to be sequences Assem.13287.C1, Assem.8702.C1, and Assem.3776.C1 (Table 3). These sequences exhibited identity to unclassified positive-strand RNA viruses. Specifically, Assem.13287.C1 exhibited identity with the helicase region (ATPase chaperone functionality) of Kelp fly virus [32] and Assem.8702.C1 exhibited identity with the 3C-like protease region of polyprotein 1 (5′-proximal ORF) of Solenopsis invicta virus 3 (SINV-3) [4]. Assem3776.C1 also exhibited identity with SINV-3 at the interface of the 3C-protease and RNA-dependent RNA polymerase. Oligonucleotide primers designed to these sequences failed to yield an amplicon by PCR (DNA template) among field-collected *N. pubens* colonies. However, 50 to 66% of field-collected colonies did generate an amplicon of anticipated size by RT-PCR with oligonucleotide primers designed to each sequence. The replicative form of the genome (minus or negative strand) was detected in a small percentage of field-collected *N. pubens* providing strong evidence that these sequences correspond to positive-strand RNA viruses that infect *N. pubens*. All of these sequences exhibited identity to viruses that infect arthropods (Diptera and Hymenoptera). Thus, detection of Assem.13287.C1, Assem.8702.C1, and Assem.3776.C1 by tagged-RT-PCR among a small percentage of field-collected *N. pubens* colonies suggests that these sequences are of viral origin and appear to replicate in *N. pubens*. Sanger sequencing of these amplicons verified their identity. Although not used as part of our screening method, host gene induction in response to viral infection can also be employed to identify viral infections indirectly. Indeed, a number of virus-induced genes/pathways were detected in the *N. pubens* transcriptome, including Toll like receptors, STAT transcription factor and Jak kinase. However, V*ago*, an important participant in the control of viral load in *Drosophila*, was not detected [33].

Pyrosequencing of entomological samples in an effort to discover viruses [7] and other organisms [34] for use as insect microbial control agents has proven an efficient method. For example, searches for virus infections in *S. invicta* by traditional means were conducted for decades without a single discovery [35], [36], [37], [38] which ultimately delayed the available use of viruses as natural enemies to control the ant. However, using the metagenomics approach, three RNA viruses were discovered in *S. invicta* which offer a new approach to control these ants [39]. Pyrosequencing of the *N. pubens* transcriptome from numerous colony sources collected from different locations throughout the state of Florida has, similarly, identified 51 sequences of putative viral origin; at least three of these sequences appear to be from virus sources that replicate in *N. pubens*. Efforts to acquire additional genome sequence and characterize the biology and impact of these potential viruses have begun in earnest.

Although the primary objective of the *N. pubens* pyrosequencing project was discovery of viruses for use in controlling this ant, the transcriptome of this pest ant was also elucidated and is now available publicly. This genetic resource will prove helpful in many future studies to understand and control, *N. pubens*, especially at this incipient stage of its expanding U.S. infestation. The *N. pubens* transcriptome represents the 7^th^ ant species (and only the 2^nd^ species in the Formicinae) to undergo a large sequencing project. These data will facilitate comparative genomic studies with the genomes of *Camponotus floridanus* (Formicinae), *Harpegnathos saltator* (Ponerinae), *Solenopsis invicta* (Myrmicinae), *Pogonomyrmex barbatus* (Myrmicinae), *Linepithema humile* (Dolichoderinae), and *Atta cephalotes* (Myrmicinae) [24], [40], [41], [42], [43].

## Supporting Information

Table S1 ***N. pubens*** **transcriptome sequences yielding a non-significant (> 1e^−4^) expectation score from BLAST analysis with viral identity.** Each sequence designation, corresponding BLAST results (gene and virus relatedness), and characteristics (nucleic acid composition, family and host range) are provided. DNA viruses are consolidated in the upper panel and RNA viruses in the lower panel.
(DOCX)
