# Supplementary material for: Metatranscriptomics and Pyrosequencing Facilitate Discovery of Potential Viral Natural Enemies of the Invasive Caribbean Crazy Ant, Nylanderia pubens
Source: PLoS One. 2012 Feb 27;7(2):e31828. doi: 10.1371/journal.pone.0031828 (PMC3288052; doi:10.1371/journal.pone.0031828)
Supplement: Table S1 — N. pubens transcriptome sequences yielding a non-significant (> 1e−4) expectation score from BLAST analysis with viral identity. Each sequence designation, corresponding BLAST results (gene and virus relatedness), and characteristics (nucleic acid composition, family and host range) are provided. DNA viruses are consolidated in the upper panel and RNA viruses in the lower panel. (DOCX) [file pone.0031828.s001.docx]

**Supplementary Table 1**

| **Designation** | **Gene** | **Virus** | **Genome** | **Family** | **Host range** |
| --- | --- | --- | --- | --- | --- |
| G49287O01AWFAF | ORF B | Autographa californica nucleopolyhedrovirus | DNA | Baculoviridae | Invertebrates |
| G49287O01DFUS7 | P48 protein | Hyphantria cunea nucleopolyhedrosis virus | DNA | Baculoviridae | Invertebrates |
| G49287O02HWFR4 | Alkaline exonuclease | Spodoptera litura nucleopolyhedrosis virus | DNA | Baculoviridae | Invertebrates |
| Ant_454Assem.12296.C1 | Transcriptional regulator | African cassava mosaic virus | DNA | Geminiviridae | Plants |
| Ant_454Assem.14461.C1 | Rh171 protein | Macacine herpesvirus | DNA | Herpesviridae | Vertebrates |
| G49287O02J3O9A | Envelope glycoprotein I | Cercopithecine herpesvirus | DNA | Herpesviridae | Vertebrates |
| Ant_454Assem.12328.C1 | 209R protein | Invertebrate iridescent virus | DNA | Iridoviridae | Invertebrates |
| Ant_454Assem.14569.C1 | Hypothetical protein | Acidianus filamentous virus | DNA | Lipothrixviridae | Bacteria |
| G49287O02JIIJ2 | Hypothetical protein | Acanothameoba polyphaga mimivirus | DNA | Mimiviridae | Protists |
| Ant_454Assem.11413.C1 | Capsid protein 3 | Acanothameoba polyphaga mimivirus | DNA | Mimiviridae | Protists |
| Ant_454Assem.1092.C9 | Hypothetical protein | Cafeteria roenbergensis virus | DNA | Mimiviridae | Protists |
| G49287O01AX18H | Ankyrin containing protein | Acanothameoba polyphaga mimivirus | DNA | Mimiviridae | Protists |
| Ant_454Assem.6902.C1 | Hypothetical protein | Acanothameoba polyphaga mimivirus | DNA | Mimiviridae | Protists |
| G49287O01B7ODO | Hypothetical protein | Human papilloma virus | DNA | Papillomaviridae | Vertebrates |
| G49287O01CU37T | Hypothetical protein | Bathycoccus virus | DNA | Phycodnaviridae | Algae |
| Ant_454Assem.8388.C1 | Membrane protein | Emiliania huxleyi virus | DNA | Phycodnaviridae | Algae |
| G49287O02GG4MT | Protein FR483_n813L | Paramecium busaria Chlorella virus | DNA | Phycodnaviridae | Algae |
| Ant_454Assem.16928.C1 | 128L protein | Yaba-like diseas virus | DNA | Poxviridae | Vertebrates/Invertebrates |
| G49287O01EE8N9 | m44R protein | Myxoma virus | DNA | Poxviridae | Vertebrates/Invertebrates |
| G49287O02I86U7 | ORF MSV011 | Melanoplus sanguinipes entomopoxvirus | DNA | Poxviridae | Vertebrates/Invertebrates |
| G49287O01AKMD8 | Ankyrin repeat protein | Canarypox virus | DNA | Poxviridae | Vertebrates/Invertebrates |
| Ant_454Assem.9380.C1 | Kelch-like protein | Sheeppox virus | DNA | Poxviridae | Vertebrates/Invertebrates |
| G49287O02IW3K3 | MPXV-WRAIR169 | Monkeypox virus | DNA | Poxviridae | Vertebrates/Invertebrates |
| G49287O02FJ38J | ATP-binding transporter | Amsacta moorei entomopoxvirus | DNA | Poxviridae | Vertebrates/Invertebrates |
| Ant_454Assem.13184.C1 | Envelope glycoprotein | Human immunodeficiency virus | RNA | Retroviridae | Vertebrates |
| G49287O01EM9OB | Envelope glycoprotein | Human immunodeficiency virus | RNA | Retroviridae | Vertebrates |
| Ant_454Assem.7192.C1 | Envelope glycoprotein | Human immunodeficiency virus | RNA | Retroviridae | Vertebrates |
| G49287O02FJKT1 | Envelope glycoprotein | Human immunodeficiency virus | RNA | Retroviridae | Vertebrates |
| G49287O02JD7GI | structural polyprotein | Venezuelan equine encephalitis virus | RNA | Togaviridae | Vertebrates |
| G49287O01CKP6I | RNA-dependent RNA polymerase | Nyamanini virus | RNA | Unassigned | Seabirds, ticks |
| G49287O02JN2F3 | RNA-dependent RNA polymerase | Nyamanini virus | RNA | Unassigned | Seabirds, ticks |
